# Indications for Autologous and Allogeneic Hematopoietic Stem-Cell Transplantation in Adults: State of the Art

**DOI:** 10.3390/jcm15176520

**Published:** 2026-08-23

**Authors:** Andrea Duminuco, Giuseppe A. Palumbo, Eleonora Avella, Alessandra Cupri, Bruno Garibaldi, Miriana Carmela Limoli, Elisa Mauro, Simona Patti, Nicolò Risata, Serena Romano, Flavia Schillaci, Andrea Spadaro, Salvatore Leotta

**Affiliations:** 1 Department of Scienze Mediche Chirurgiche e Tecnologie Avanzate “G.F. Ingrassia”, University of Catania, 95123 Catania, Italy; 2 Division of Hematology with BMT, A.O.U. Policlinico “G.Rodolico-San Marco”, Via S. Sofia 78, 95123 Catania, Italy; eleonora.avella62@gmail.com (E.A.); alessandracupri@hotmail.it (A.C.); brunga93@gmail.com (B.G.); mirianalimoli@virgilio.it (M.C.L.); elixmauro@hotmail.it (E.M.); risatanico59@gmail.com (N.R.); serenaromano54@gmail.com (S.R.); flavia.schilla@gmail.com (F.S.); andreaspadaro@hotmail.it (A.S.); leotta115@gmail.com (S.L.)

**Keywords:** allogeneic HSCT, autologous HSCT, acute leukemia, myelodysplastic syndromes, lymphoma, multiple myeloma, myelofibrosis, aplastic anemia, measurable residual disease, graft-versus-host disease

## Abstract

Hematopoietic stem-cell transplantation (HSCT) has evolved from a salvage procedure for otherwise-fatal leukemia into a curative modality spanning nearly every hematological malignancy and several non-malignant disorders. The contemporary landscape has been reshaped by three converging forces: the refinement of disease-specific risk stratification (European LeukemiaNet [ELN] 2022 for acute myeloid leukemia, Molecular International Prognostic Scoring System [IPSS-M] for myelodysplastic syndromes, Mutation-Enhanced International Prognostic Scoring System [MIPSS70+ v2.0], and Myelofibrosis Transplant Scoring System [MTSS] for myelofibrosis); the integration of measurable residual disease (MRD) into dynamic, response-adapted transplant decisions; and the approval of immune effector cell therapies that have displaced transplantation from several long-standing indications while creating new ones (bridge-to-transplant, post-CAR-T consolidation). In parallel, post-transplant cyclophosphamide (PTCy) has largely equalized outcomes across matched sibling, matched unrelated, and mismatched alternative donors, and novel agents (ruxolitinib, belumosudil, axatilimab) have materially reduced graft-versus-host disease (GVHD) morbidity. This narrative review synthesizes current indications for autologous (auto-HSCT) and allogeneic (allo-HSCT) transplantations in adults, and flags areas of persistent controversy where randomized data are still maturing. Across all indications, the clinician’s question is shifting from “transplant or not?” towards “which donor, which conditioning, which bridge, and which post-transplant maintenance?”, each tailored to disease biology, MRD trajectory, and patient fitness.

## 1. Introduction

### 1.1. Historical Background and Rationale

The modern era of hematopoietic stem-cell transplantation (HSCT) opened with the syngeneic and allogeneic bone marrow grafts performed by E. Donnall Thomas and colleagues in Seattle between 1957 and 1969, which established that marrow grafting could cure advanced leukemia, earning the Nobel Prize in 1990 [1,2]. Over the subsequent five decades, the field expanded from HLA-identical sibling grafts to encompass matched unrelated donors (MUDs), umbilical cord blood, mismatched unrelated donors (MMUDs) and, most importantly, haploidentical family donors using PTCy-based GVHD prophylaxis, an approach pioneered at Johns Hopkins that has since become standard across donor types [3].

If auto-HSCT is essentially a high-dose chemotherapy procedure in which the graft rescues marrow function but carries no anti-tumor immunity, allo-HSCT adds a donor-derived immune component that mediates the graft-versus-leukemia/lymphoma (GVL) effect, at the price of GVHD and infection. Conditioning is classified as myeloablative (MAC), reduced-intensity (RIC), or non-myeloablative (NMA) according to consensus criteria [4]. Peripheral blood stem cells (PBSCs) have largely replaced bone marrow as the adult allograft source because of faster engraftment, although bone marrow retains an advantage in chronic GVHD in the unrelated-donor setting [5], and in the haploidentical setting with PTCy [6]. Cord blood use has declined in adults, but remains relevant for patients lacking other donors, with ex vivo expanded products such as omidubicel mitigating the historical limitation of delayed engraftment [7].

### 1.2. Donor Selection in the Current Era

The classical hierarchy (HLA-identical sibling first, 10/10 MUD second, alternative donor third) has been substantially flattened [8]. The BMT CTN 1703 randomized trial demonstrated the superiority of PTCy/tacrolimus/mycophenolate over tacrolimus/methotrexate as GVHD prophylaxis in reduced-intensity allo-HSCT from matched donors, with one-year GVHD-free, relapse-free survival (GRFS) of 52.7% versus 34.9% (hazard ratio [HR]: 0.64, 95% CI: 0.49–0.83) [9]. An initial CIBMTR registry analysis reported worse overall survival with haploidentical compared with matched unrelated donor transplantation in the RIC setting [10], although a subsequent propensity-score-matched re-analysis of the same dataset found no significant difference in overall survival between the two donor types [11]. Prospective phase II data have supported PTCy-based MMUD bone marrow transplantation [12], and this rationale has been substantially reinforced by a large CIBMTR comparison showing that PTCy attenuates the disadvantage of HLA-mismatched unrelated donor transplantation and by the BMT CTN 1702 prospective trial confirming the safety and efficacy of PTCy-based mismatched unrelated donor transplantation in a multicenter phase II setting [13,14]. Consistent with these data, the 2025 EBMT practice recommendations now group cord blood, haploidentical, and mismatched unrelated donors into a single “mismatched alternative donor” (MMAD) category [15].

Reflecting this convergence, the 2025 EBMT practice recommendations now group cord blood, haploidentical, and mismatched unrelated donors into a single “mismatched alternative donor” (MMAD) category, distinct from well-matched related and unrelated donors [15]. In practice, donor selection is driven by HLA matching, donor age (younger preferred), CMV serostatus, sex mismatch (female to male associated with higher chronic GVHD risk), absence of donor-specific anti-HLA antibodies in the haploidentical setting, and graft availability and timing.

**Figure 1:**
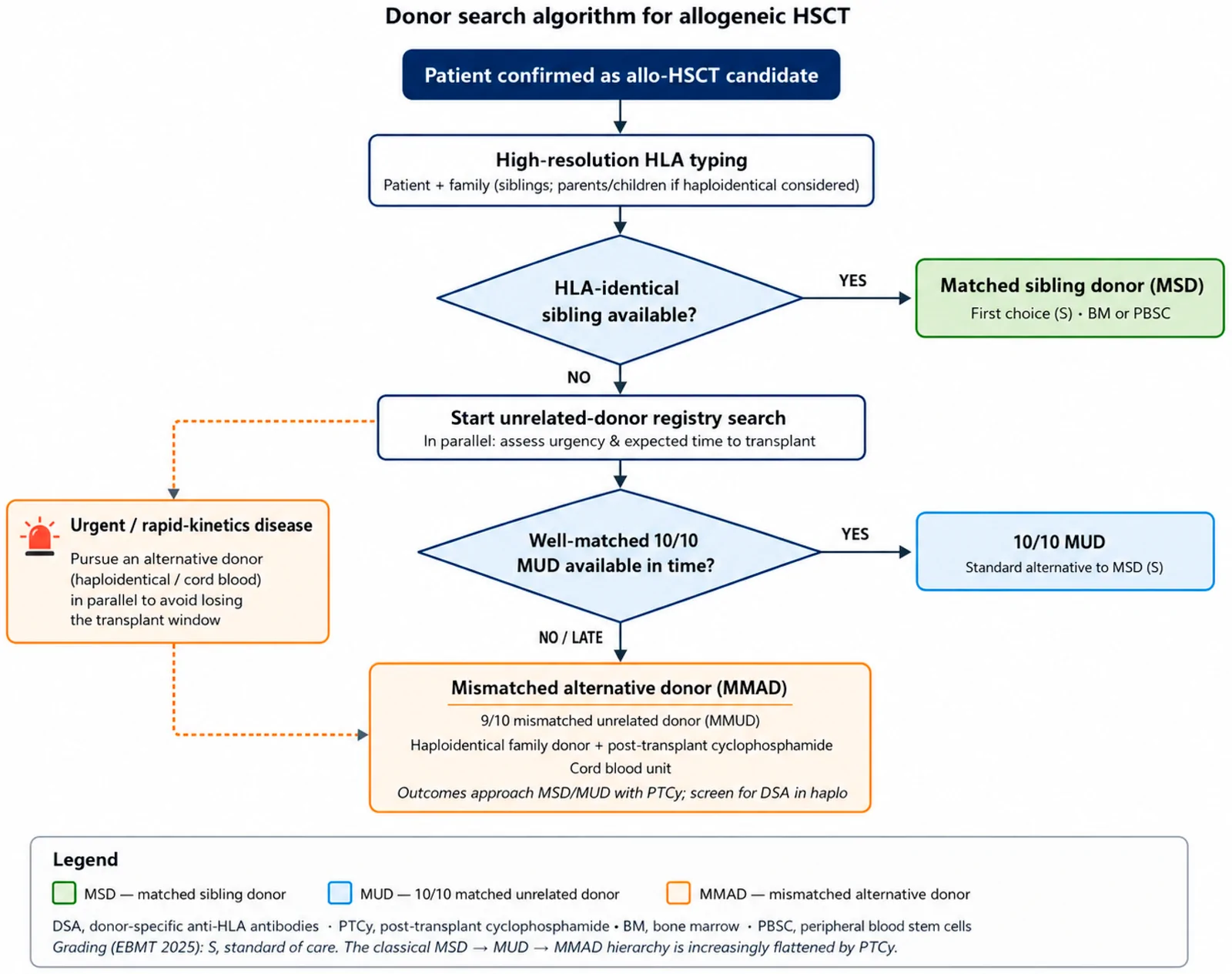
Contemporary donor-search algorithm for allogeneic hematopoietic stem-cell transplantation. Once a patient is confirmed as a transplant candidate, high-resolution HLA typing of the patient and family is started in parallel with an unrelated-donor registry search when no HLA-identical sibling is available. The traditional hierarchy—matched sibling donor (MSD) → 10/10 matched unrelated donor (MUD) → mismatched alternative donor (MMAD: 9/10 mismatched unrelated, haploidentical with post-transplant cyclophosphamide, or cord blood)—has been flattened by post-transplant cyclophosphamide, which yields comparable outcomes across donor types. In urgent disease, an alternative donor pathway is pursued in parallel to avoid missing the transplant window. DSAs, donor-specific anti-HLA antibodies; PTCy, post-transplant cyclophosphamide.

Because PTCy-based haploidentical and mismatched-donor transplantation now achieves outcomes approaching those of matched transplants, virtually every eligible patient has a donor, and the practical questions have become the speed and sequencing of the search rather than its feasibility. As soon as a patient is deemed a transplant candidate, high-resolution HLA typing of the patient and potential family donors (siblings, and, where a haploidentical donor is contemplated, parents and children) should be initiated, together with a parallel search of the unrelated-donor registry whenever no HLA-identical sibling is available. In diseases with rapid kinetics or a narrow window for transplantation, an alternative donor (haploidentical or cord blood) should be pursued in parallel to avoid missing the optimal transplant window while awaiting confirmation of an unrelated donor. The overall process is summarized in Figure 1.

### 1.3. Conditioning Regimens

Standard MAC regimens include busulfan–cyclophosphamide (BuCy) and 12 Gy total-body irradiation with cyclophosphamide (TBI12–Cy). Over the past two decades, these have been progressively replaced in clinical practice by less-toxic myeloablative platforms such as busulfan (12.8 mg/kg)–fludarabine (BuFlu), fludarabine–TBI (Flu-TBI 12 Gy), and treosulfan-based regimens, the latter shown to reduce non-relapse mortality without loss of disease control [16,17,18]. These reduced-toxicity regimens have lowered transplant-related mortality (TRM) while preserving anti-leukemic activity, and RIC platforms (fludarabine–melphalan, reduced-dose fludarabine–busulfan, treosulfan–fludarabine FT10) have extended eligibility into the seventh decade of life [19].

In the BMT CTN 0901 trial (AML/MDS), myeloablative conditioning improved relapse-free survival compared with RIC in the overall cohort, whereas the overall survival advantage did not reach statistical significance in the whole population and emerged only in the AML subgroup [19]. A subsequent MRD-driven analysis of the same trial showed that the survival benefit of myeloablative conditioning was largely concentrated in patients with detectable pre-transplant MRD, providing one of the strongest biological rationales for MRD-guided conditioning intensity in AML patients [20].

Conversely, the RICMAC trial, which predominantly enrolled patients with MDS and a smaller proportion of secondary AML, showed a trend towards a survival advantage for RIC with comparable relapse incidence between arms [21]. For patients transplanted with active disease, sequential conditioning regimens such as FLAMSA-RIC (fludarabine, amsacrine, cytarabine followed by reduced-intensity TBI/cyclophosphamide), designed two decades ago for active acute leukemia, remain a preferred option [22,23].

### 1.4. Aim and Scope

The objective of this narrative review is to provide a concise, evidence-graded overview of HSCT indications in adult hematology, integrating standard baseline treatments and contemporary guideline statements with pivotal trial data. A synopsis of adult indications by disease and disease status, graded with the EBMT four-tier system, is provided in Table 1.

## 2. Acute Myeloid Leukemia

### 2.1. Disease Overview and Conventional Treatment

AML accounts for roughly 80% of adult acute leukemias, with age-adjusted incidence rising steeply after the age of 60 years, and it represents the single most common indication for allo-HSCT, comprising approximately 40% of allografts [15,24].

The 2022 ELN classification stratifies patients by cytogenetic and molecular markers into favorable, intermediate, and adverse risk groups. Key revisions from ELN 2017 include the reclassification of FLT3-ITD as intermediate risk irrespective of allelic ratio or co-occurring NPM1 mutation (a change that real-world survival data increasingly challenge), the inclusion of myelodysplasia-related gene mutations (*ASXL1*, *BCOR*, *EZH2*, *RUNX1*, *SF3B1*, *SRSF2*, *STAG2*, *U2AF1*, *ZRSR2*) in the adverse category, and the downgrading of TP53 mutations with variant allele frequency ≥10% to adverse risk [25,26]. Concurrent MRD assessment by multiparameter flow cytometry and/or by RT-qPCR for validated molecular markers (NPM1, RUNX1::RUNX1T1, CBFB::MYH11, PML::RARA) is mandatory for risk-adapted decision-making [27,28].

Standard induction for fit adults remains “7+3” (continuous-infusion cytarabine in the range of 100–200 mg/m^2^/day for 7 days plus an anthracycline: daunorubicin in the range of 60–90 mg/m^2^, idarubicin at 12 mg/m^2^, or mitoxantrone at 12 mg/m^2^ for 3 days) [29]. Midostaurin is added for FLT3-mutated disease [30,31,32], quizartinib in combination with “7+3” chemotherapy is a further approved option for FLT3-ITD-mutated AML, with the QUANTUM-First trial demonstrating a significant overall survival benefit over placebo plus chemotherapy [33]. Gemtuzumab ozogamicin is added for CD33-positive favorable/intermediate-risk disease [34]. In secondary or therapy-related AML, CPX-351 (liposomal daunorubicin/cytarabine) confers a survival advantage over “7+3” [35,36]. For patients unfit for intensive chemotherapy, azacitidine plus venetoclax achieves composite complete remission (CR) rates of approximately 66% and a median OS of 14.7 versus 9.6 months for azacitidine alone, and is now the frontline standard in this population [37,38,39,40]. Post-remission consolidation for fit patients is generally three to four cycles of high-dose cytarabine (3 g/m^2^ every 12 h on days 1, 3, and 5) [41,42]. More recently, intermediate-dose cytarabine (1.5 g/m^2^ every 12 h) has emerged as a comparably effective option with reduced toxicity, supported by the ALFA-1200 study demonstrating non-inferiority to the 3 g/m^2^ dose in patients aged ≤ 60 years [43].

### 2.2. Allo-HSCT in First-Complete-Remission Patients

The decision to proceed to allo-HSCT in AML patients is governed by the balance between relapse risk (estimated from genetics, response kinetics, and post-induction MRD) and the predicted non-relapse mortality of the procedure, the latter captured by comorbidity scores such as the HCT-CI and the Disease-Risk Index [44,45].

For adverse-risk AML cases, allo-HSCT in CR1 from any suitable donor is the standard of care in eligible patients, with a five-year OS in the range of approximately 40–50% versus 10–20% with chemotherapy alone [46]. For intermediate-risk AML cases, the indication is individualized and MRD-driven. Patients with detectable MRD after induction or first consolidation have a strong indication, whereas MRD-negative patients may be transplanted from a matched sibling, with alternative-donor transplantation requiring careful risk–benefit discussion [47,48].

For favorable-risk AML cases (core-binding-factor AML with sustained MRD clearance, and *NPM1*-mutated/*FLT3*-ITD-negative or *CEBPA*-mutated AML with MRD clearance), allo-HSCT in CR1 is generally not recommended. High-dose cytarabine consolidation alone yields long-term OS in the range of 60–70%, and transplant is reserved for MRD persistence, molecular relapse, or CR2 [48].

However, in core-binding-factor AML that fails to achieve a 3-log reduction in fusion transcript after two cycles, and in NPM1-mutated AML that remains MRD-positive after two cycles, the relapse risk is high, and allo-HSCT is beneficial [49,50].

Therapy-related and secondary AML patients generally retain an adverse-risk indication for CR1 transplant [51,52].

Haploidentical allo-HSCT is considered the standard of care for secondary AML [15]. An important exception is *t*-AML carrying an isolated *NPM1* mutation, which behaves analogously to de novo NPM1-mutated AML and may be managed without upfront allo-HSCT if MRD is cleared [53].

### 2.3. MRD-Driven Decision-Making

MRD status at the time of transplant is among the strongest predictors of outcome [54]. The pre-transplant Measurable Residual Disease (Pre-MEASURE) study, analyzing 1075 adults with NPM1- or FLT3-ITD-mutated AML transplanted in first remission, found that persistent MRD by ultra-deep next-generation sequencing was associated with three-year relapse of 68% versus 21% and OS of 39% versus 63% relative to MRD-negative patients [55]. Where feasible, therefore, induction and consolidation now aim for MRD-negativity before transplant, with venetoclax-containing regimens (e.g., FLAG-IDA–venetoclax) increasingly deployed as bridging in MRD-positive or refractory disease. After transplant, serial MRD monitoring at months 1, 3, 6, 9, and 12 is routine, and pre-emptive azacitidine can avert overt relapse in patients with rising MRD, as shown in the RELAZA2 trial [56]. In FLT3-mutated disease, post-transplant maintenance improves outcomes: sorafenib reduced relapse and improved leukemia-free survival in the SORMAIN trial [57], and gilteritinib maintenance was supported by the phase III MORPHO trial, with benefits concentrated in MRD-positive patients [58].

### 2.4. Allo-HSCT in FLT3-ITD-Mutated AML Patients in First Complete Remission

FLT3-mutated AML has historically carried a worse prognosis than FLT3-wild-type disease [59]. Yet FLT3-mutated AML is biologically heterogeneous and spans several risk classes depending on co-mutations in *NPM1*, *ASXL1*, *TP53*, and *RUNX1* [26]. Improved understanding of disease biology and the introduction of potent FLT3 inhibitors have reopened the question of whether every eligible patient with a FLT3 mutation in MRD-negative CR1 should proceed to allo-HSCT [60]. The debate is complicated by the marked heterogeneity of the source studies, which makes uniform translation into individual-centered practice difficult.

A combined analysis of the UK NCRI AML17 and AML19 trials found no survival benefit from allo-HSCT in CR1 for NPM1-mutated/FLT3-ITD patients who were MRD-negative [61]. Similarly, a post hoc analysis of the CALGB 10603/RATIFY trial showed that midostaurin benefited all three ELN 2017 risk groups, whereas allo-HSCT in CR1 conferred a survival advantage only in adverse-risk patients [62]. Taken together, these data support withholding transplant in CR1 from MRD-negative NPM1/FLT3-ITD patients.

Several caveats, however, temper the generalizability of this conclusion. First, the induction and consolidation backbones in these trials were heterogeneous: CPX-351 and FLAG-IDA in AML19, cytarabine/daunorubicin/etoposide with gemtuzumab ozogamicin and a lestaurtinib randomization in AML17, and 200 mg/m^2^ of cytarabine (days 1–7) with high-dose cytarabine consolidation plus 12 months of midostaurin maintenance in RATIFY [30,63,64]. In routine practice, such regimens (in particular, FLT3-inhibitor maintenance) are not uniformly delivered. Second, FLT3-ITD AML is a multiclonal disease, and MRD-negativity assessed on markers other than FLT3-ITD does not guarantee the eradication of all clones; sensitive FLT3-ITD MRD by NGS remains beyond the reach of many centers [58]. Third, the approval of quizartinib during induction/consolidation and as maintenance, based on QUANTUM-First, will further reshape transplant decisions. Notably, the highest survival in that trial was seen among quizartinib-arm patients who underwent allo-HSCT in CR1 [33,65].

In summary, transplant decisions in FLT3-mutated AML cases must be made against a rapidly evolving therapeutic backdrop. EBMT 2025 recommends allo-HSCT in CR1 for patients with either both wild-type or both mutated NPM1 and FLT3-ITD, whereas the NCCN guidelines list allo-HSCT as “preferred” for eligible FLT3-ITD patients in CR1 from a conventional or alternative donor. MRD-negative CR1 patients with a high transplant risk and/or without a well-matched donor may reasonably defer transplant in favor of close MRD monitoring, proceeding to pre-emptive transplant only on molecular recurrence.

### 2.5. Relapsed/Refractory AML and Second Transplant

For primary refractory or relapsed AML cases, achieving CR2 is a prerequisite for consolidation with allo-HSCT. Salvage options include FLAG-IDA, MEC, CLAG-M, and, increasingly, venetoclax-based combinations. For patients unable to achieve CR2, sequential conditioning (FLAMSA-RIC) permits transplantation with active disease at a five-year OS roughly in the range of 25–30% [22], and allo-HSCT retains curative potential in fit primary refractory patients when a donor can be identified rapidly and predicted TRM is acceptable [15]. A second allo-HSCT for post-transplant relapse is feasible in selected patients. EBMT registry data indicate that time from first transplant to relapse >12 months, Karnofsky performance status ≥80%, and a change in donor independently favor the outcome, with two-year OS in the range of around 25–30% [66]. Patients with truly resistant disease should preferentially be enrolled in clinical trials.

### 2.6. Acute Promyelocytic Leukemia

In acute promyelocytic leukemia (APL), frontline all-trans retinoic acid plus arsenic trioxide (with or without idarubicin, depending on the baseline white-cell count) cures the vast majority of patients, with MRD monitored by quantitative PML::RARA PCR. Allo-HSCT is not indicated in CR1 or in molecular CR2. In molecular (MRD-positive) CR2 or primary refractory disease, auto-HSCT following harvest of a PML::RARA-negative graft is equivalent to matched-sibling allo-HSCT and superior to alternative-donor allo-HSCT [67,68,69].

## 3. Acute Lymphoblastic Leukemia

### 3.1. Risk Stratification and Conventional Therapy

ALL is the second-most-common indication for allo-HSCT, accounting for approximately 16% of allografts [70]. Adult ALL is stratified by age, white-cell count, immunophenotype, cytogenetic/molecular features, and, above all, MRD response. Adverse features include age > 35 years, white-cell count >30 × 10^9^/L (B-ALL) or >100 × 10^9^/L (T-ALL), pro-B immunophenotype, *KMT2A* rearrangement, low hypodiploidy or near-triploidy, complex karyotype (≥5 abnormalities), *TP53* mutation, Ph-like genotype, early T-cell precursor immunophenotype, and failure to achieve CR after one induction cycle [71]. Pediatric-inspired regimens (GIMEMA LAL1913, GRAALL-2005, CALGB 10403) are now standard up to the age range of 55–65 years [72]. The MRD, the single-most-important prognostic factor, is measured by multiparameter flow cytometry (threshold 10^−4^) or molecular methods (IGH/TR rearrangements by NGS, BCR::ABL1 qPCR for Ph-positive disease), and now anchors the transplant decision in most study groups [73].

Two randomized trials have transformed frontline management. In ECOG-ACRIN E1910, adults with Ph-negative B-ALL in MRD-negative CR1 who received blinatumomab in addition to consolidation had a three-year OS of 83% versus 65% for consolidation alone (HR: 0.41, 95% CI: 0.23–0.73), establishing blinatumomab consolidation as a new standard [74]. In the GIMEMA D-ALBA study, a chemotherapy-free induction of dasatinib plus blinatumomab in newly diagnosed Ph-positive ALL cases achieved molecular CR in roughly 60% of patients, with durable long-term survival [75,76]. This chemotherapy-free paradigm has been further refined with third-generation TKIs: the phase II MDACC study of ponatinib plus blinatumomab in newly diagnosed Ph-positive B-ALL cases reported a complete molecular response rate of approximately 85% and encouraged early survival, without upfront intensive chemotherapy or protocol-mandated allo-HSCT [77]. In this context, the historical CR1 indication for allo-HSCT in Ph-positive ALL cases is challenged, as patients achieving a deep molecular response may enjoy durable remission without transplant [78].

### 3.2. Allo-HSCT Indications

Allo-HSCT in CR1 is the standard of care for high-risk Ph-negative B- and T-ALL, for any adult with molecular failure (MRD > 10^−4^ after three consolidation blocks), and for Ph-positive ALL with MRD persistence after TKI-based induction [79]. Allo-HSCT should also be considered in ALL individuals with adverse cytogenetics at diagnosis (low hypodiploidy, KMT2A rearrangement, t(8;14), complex karyotype), particularly when combined with a suboptimal MRD response. However, data from the GRAALL group suggest that low hypodiploidy and complex karyotype may lose their historical adverse prognostic weight in adults treated with pediatric-inspired intensive regimens, and therefore may not per se mandate allo-HSCT in MRD-negative first CR cases [80]. Conversely, standard-risk Ph-negative ALL with MRD clearance carries no CR1 transplant indication, and Ph-positive ALL in MRD-negative CR1 after modern chemotherapy-free or TKI-plus-antibody regimens may increasingly be managed without transplant. The optimal timing for MRD assessment in Ph-positive B-ALL cases is currently regarded as day +90 after the initiation of therapy, approximately corresponding to the end of consolidation: in a retrospective analysis of 230 adults with Ph-positive B-ALL enrolled on modern regimens, achievement of a complete molecular response by day +90 was associated with excellent long-term outcomes irrespective of transplant, whereas patients with detectable BCR::ABL1 at that landmark derived clear benefit from consolidative allo-HSCT [81]. Day-90 MRD assessment is therefore emerging as a key decisional landmark for transplant allocation in this setting. Beyond CR1, allo-HSCT is the standard for any patient achieving CR2, from any available donor, including non-T-cell-depleted haploidentical donors [82]. In T-ALL, where salvage options are limited, consolidation with allo-HSCT in CR1 should be considered for high-risk patients defined by MRD, early T-cell precursor immunophenotype, and adverse cytogenetics [73,83].

The choice of conditioning is age- and disease-dependent. In children, adolescents, and young adults (up to ~21 years), the FORUM trial demonstrated that 12 Gy TBI-based myeloablative conditioning is superior to chemotherapy-only myeloablative conditioning in terms of both overall survival and relapse, and it is currently regarded as the standard [84]. In older adults, and specifically in Ph-positive ALL, registry data indicate comparable overall survival between myeloablative and reduced-intensity conditioning, so RIC represents a valid option for patients unfit for full-intensity myeloablation [85]. Thiotepa- or busulfan-based platforms are alternatives when TBI is unavailable or contraindicated. Before proceeding to transplant, patients with persistent or recurrent MRD should switch therapy. Blinatumomab is the only agent proven to convert MRD-positive Ph-negative B-ALL to MRD-negativity (in approximately 78%) with a survival benefit, and serves effectively as a bridge to allo-HSCT [86].

### 3.3. CAR-T Integration and Second Transplants

Immunotherapy has revolutionized the relapsed/refractory B-ALL landscape. Blinatumomab and inotuzumab ozogamicin achieve deep remissions as bridges to transplant, while CD19 CAR-T is approved for patients up to 25 years of age (tisagenlecleucelbased on ELIANA) and for adults (brexucabtagene autoleucel, per ZUMA-3), and obecabtagene autoleucel, per FELIX [87,88,89]. More than 70% of patients achieve CR with CAR-T regardless of cytogenetics or prior therapy, though responses may be inferior after prior blinatumomab, and toxicity may be higher after prior allo-HSCT [90]. Relapse after CAR-T occurs in 30–50% of patients (up to 40% CD19-negative). The role of consolidative allo-HSCT after CAR-T remains unsettled. Current guidance suggests that patients with molecular MRD-positivity after CAR-T, loss of B-cell aplasia, and no prior transplant are candidates for consolidative allo-HSCT [15]. A second allo-HSCT for post-transplant relapse is rarely successful with chemotherapy alone. The preferred contemporary approach is CAR-T as a bridge, followed, where appropriate, by a second allograft from a different donor [91,92].

**Figure 2:**
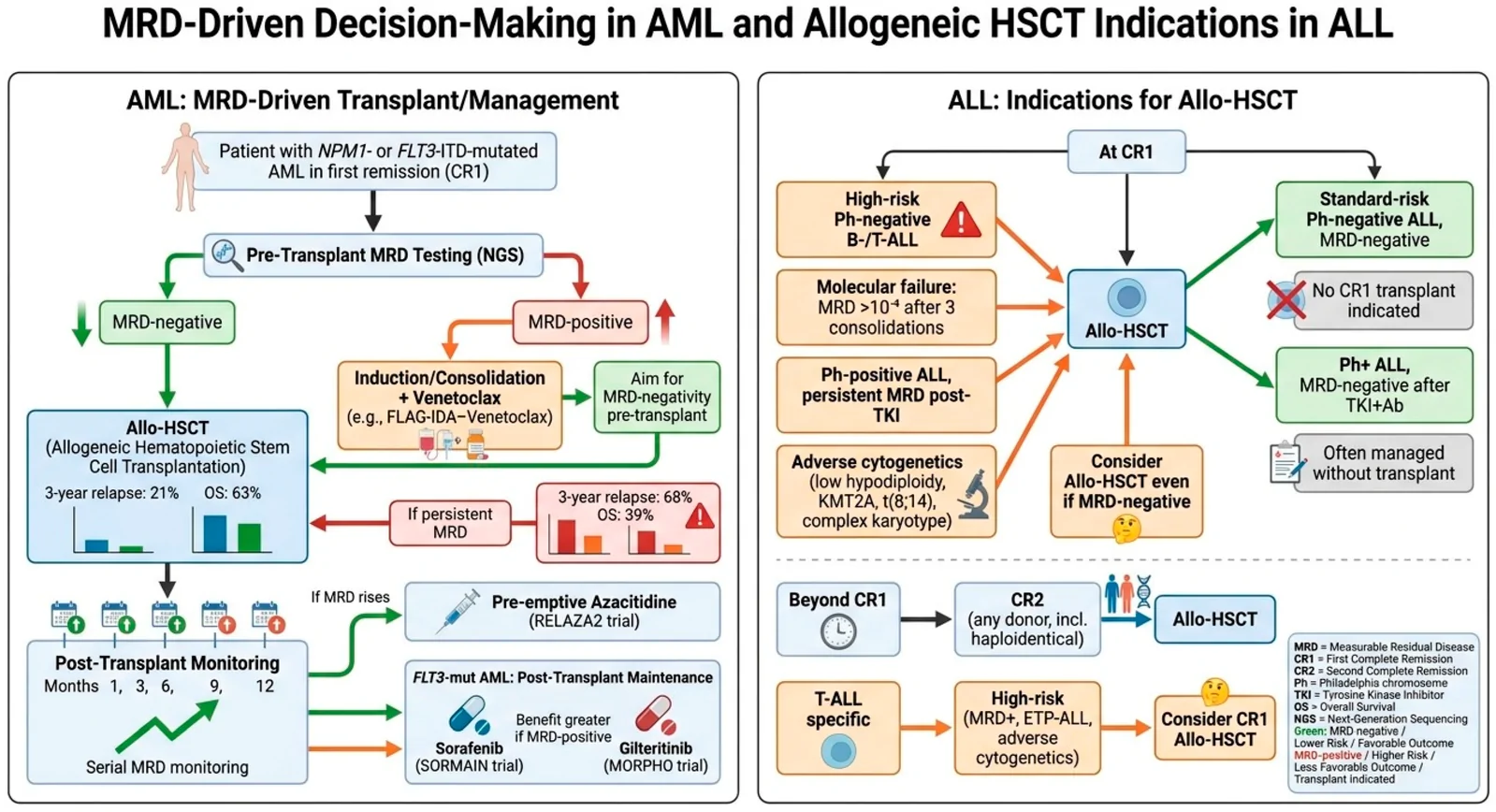
MRD-based indications for allo-HSCT in acute myeloid leukemia (AML) and acute lymphoblastic leukemia (ALL) cases, as described in the text.

Figure 2 presents a flow chart for AML and ALL indications.

## 4. Myelodysplastic Syndromes

### 4.1. Prognostic Models and Conventional Treatment

Adult MDS comprises clonal stem-cell disorders with variable risk of progression to AML [93,94]. The revised IPSS has been refined by the molecular IPSS (IPSS-M), which incorporates 31 gene mutations; reclassifies 46% of patients, most moving to higher-risk categories; and defines six risk groups from very low to very high [95]. For lower-risk disease with symptomatic anemia, erythropoiesis-stimulating agents are first-line when serum erythropoietin is <500 U/L [96]. Luspatercept improved transfusion independence relative to epoetin alfa in the COMMANDS trial and is a preferred option for ring-sideroblast-positive/SF3B1-mutated disease [97], while lenalidomide is effective in del(5q) disease [98]. For higher-risk disease, hypomethylating agents remain the backbone. The addition of venetoclax raised response rates in phase II but did not meet its survival endpoint in the phase III VERONA trial [99]. IDH1-mutated patients may benefit from ivosidenib [100].

### 4.2. Allo-HSCT Indications

Allo-HSCT remains the only curative option for MDS. The prospective donor-versus-no-donor BMT CTN 1102 trial in higher-risk MDS aged 50–75 years demonstrated three-year OS of 47.9% with allo-HSCT versus 26.6% without, and a large IPSS-M-based analysis confirmed that patients in the moderate–high, high, and very-high categories benefit from immediate transplantation, whereas lower-risk categories do not [101,102]. A post hoc IPSS-M reclassification of the BMT CTN 1102 cohort by Versluis et al. further refined this observation, showing that, among the six IPSS-M risk categories, only patients in the very-high-risk group derived a statistically significant overall survival benefit from the donor arm relative to the very-low reference, an important nuance that argues for particularly early transplant referral in very-high-risk IPSS-M patients [103].

Higher-risk MDS (IPSS-M moderate–high/high/very-high, or IPSS-R intermediate-2/high/very high) carries a standard-of-care indication for allo-HSCT in eligible patients [104].

The role of allo-HSCT in lower-risk MDS patients remains one of the most nuanced decisions in the field. As a group, patients with very low, low, or moderate–low IPSS-M disease derive no survival benefit from immediate transplantation and are best managed with supportive care, erythropoiesis-stimulating agents, luspatercept, or lenalidomide for del(5q) disease [95,97]. However, a well-defined subset of otherwise lower-risk patients acquires a transplant indication when adverse features supervene, including profound cytopenias (hemoglobin < 80 g/L, neutrophils < 0.8 × 10^9^/L, platelets < 50 × 10^9^/L), heavy transfusion dependence, secondary marrow fibrosis, bi-allelic TP53 mutation, complex or monosomal karyotype, or failure of hypomethylating and erythroid-directed therapies [105]. Consistent with this, the 2023 ASTCT MDS transplant guidelines also recognize failure of non-transplant therapies (including hypomethylating agents, erythropoiesis-stimulating agents, luspatercept, or lenalidomide for del(5q) disease) as a legitimate indication for allo-HSCT in otherwise lower-risk patients [106]. The introduction of IPSS-M has refined this decision. Approximately 46% of patients are reclassified relative to IPSS-R, and up-staging into the moderate–high category identifies patients who benefit from earlier transplantation despite an apparently indolent clinical presentation [95]. In eligible younger patients (typically <65 years) with a suitable donor, timely allo-HSCT is preferred over a delayed strategy, since disease progression and cumulative iron/transfusion-related organ damage progressively erode the transplant window. Conversely, in older patients, those with limited donor options, or when IPSS-M genomic upgrading is absent, a watch-and-wait approach with structured monitoring (including serial IPSS-M reassessment on new mutations or cytopenia progression) is preferable to upfront transplantation, whose non-relapse mortality would otherwise outweigh the modest projected survival gain.

Intermediate-risk disease with a range of 5–10% blasts remains an area of genuine controversy, with upfront transplant weighed against a hypomethylating-first strategy [107].

Pre-transplant cytoreduction has not been shown to improve outcomes. Indeed, a recent EBMT study showed that down-staging IPSS-R with pre-HCT therapy did not translate into better survival [108]. Where upfront transplant is not feasible, hypomethylating agents are used to prevent progression, and stable marrow-blast persistence is not a contraindication to transplant. As with AML, myeloablative conditioning reduces relapse in patients with <5% blasts, whereas the RICMAC trial found no OS difference in MDS [19,21]. Auto-HSCT has no role in MDS. TP53-mutated MDS/AML remains a major unmet need, with post-transplant relapse exceeding 60% regardless of conditioning intensity, motivating the study of maintenance and novel cellular strategies [107,109].

### 4.3. Chronic Myelomonocytic Leukemia

Allo-HSCT is the only potentially curative treatment for chronic myelomonocytic leukemia (CMML), but it is applicable to a minority of patients, with high relapse rates (30–50%) and non-relapse mortality (>20%) that demand careful selection [110]. Risk assessment should use a molecularly informed score such as the CPSS-Mol [111]. Patients with high-risk CPSS-Mol disease and a suitable donor should proceed directly to allo-HSCT, as should those with intermediate-2 CPSS-Mol disease plus at least one additional risk factor (extramedullary disease, marked splenomegaly, hyperleukocytosis) [110,112]. Low- and intermediate-1-risk patients are monitored dynamically rather than transplanted upfront. Upfront transplantation is preferred over pre-transplant therapy unless donor availability or aggressive kinetics dictate otherwise, in which case hypomethylating-based bridging is favored.

## 5. Myeloproliferative Neoplasms

### 5.1. Myelofibrosis

Primary and secondary myelofibrosis (MF) remain the principal HSCT indication among BCR::ABL1-negative MPN, and allo-HSCT is the only curative modality. Risk stratification has evolved from DIPSS/DIPSS-plus to mutation-enhanced scores [113]. MIPSS70+ v2.0 and the transplant-specific MTSS incorporate high-molecular-risk mutations (ASXL1, SRSF2, U2AF1 Q157, EZH2, IDH1/2) and triple-negative driver status [114,115].

Medical therapy of MF is centered on JAK inhibitors, which control splenomegaly and constitutional symptoms and modestly prolong survival, but do not eradicate the malignant clone, so that does not substitute for transplantation in eligible high-risk patients [116]. Ruxolitinib, the first-in-class JAK1/2 inhibitor, reduced spleen volume and symptom burden and conferred a survival advantage over placebo and best available therapy in the COMFORT-I and COMFORT-II trials [117,118] and remains the standard first-line agent for patients with platelets ≥50 × 10^9^/L, even if related to immunosuppressive risk and infections [119,120,121,122,123,124,125]. Fedratinib, a JAK2/FLT3 inhibitor, is active both in the frontline setting and after ruxolitinib failure (JAKARTA and JAKARTA-2) [126,127]. Momelotinib, which additionally inhibits ACVR1/ALK2 and thereby suppresses hepcidin to improve erythropoiesis, addresses the anemia that dominates the natural history of MF [128]. It improved anemia, spleen, and symptom endpoints in SIMPLIFY-1, and, in the ruxolitinib-exposed anemic population, in MOMENTUM [129,130]. Pacritinib, which spares JAK1 and inhibits IRAK1 and ACVR1, is the preferred agent for patients with severe thrombocytopenia (platelets <50 × 10^9^/L) based on PERSIST-2 [131]. Anemia-directed options (luspatercept, danazol, erythropoiesis-stimulating agents) complement JAK inhibition in transfusion-dependent patients [132].

Because the therapeutic window for transplantation is defined largely by the disease trajectory during JAK inhibition, the systematic assessment of ruxolitinib response and the early recognition of failure have become central to transplant timing. Response should be evaluated against IWG-ELN criteria, and the RR6 model (which combines ruxolitinib dose, spleen response, and transfusion requirement at six months) allows the early identification of patients destined for poor survival who may benefit from prompt reassessment for transplantation or a switch of therapy [133,134,135]. This concept underlies the argument for early intervention, for example, switching to momelotinib during the moderate-anemia phase rather than awaiting overt transfusion dependence, and for reassessing transplant eligibility at the first sign of ruxolitinib failure rather than after clinical deterioration [136]. Emerging combinations aim at disease modification [137]. The BET inhibitor pelabresib (MANIFEST-2) [138,139,140], the MDM2 inhibitor navtemadlin, selinexor, and luspatercept added to ruxolitinib are under active investigation and may, in time, further reshape the pre-transplant treatment sequence, though none has yet demonstrated a curative effect that would displace allo-HSCT [137].

Against this therapeutic backdrop, the EBMT/ELN international working group recommends allo-HSCT for eligible patients with primary MF and an intermediate-2 or high-risk DIPSS score, or high-risk MIPSS70/MIPSS70-plus, and for secondary MF with high/intermediate-2-risk MYSEC-PM, provided the MTSS score is low or intermediate [141]. Younger patients with intermediate-risk disease and a low MTSS score, particularly those harboring multi-hit *TP53*, may also be candidates after balancing patient preference, donor type, and competing options [142]. Auto-HSCT has no role in MF.

Peri-transplant management of splenomegaly is a critical determinant of outcome. Spleen size at transplant is independently associated with delayed engraftment, higher non-relapse mortality, and inferior overall survival: retrospective series have shown that a bipolar spleen length > 20–22 cm, or a palpable spleen > 5 cm below the costal margin, translates into progressively worse post-transplant outcomes [143,144,145].

Consequently, candidates with significant splenomegaly should receive spleen-directed therapy before transplant. JAK inhibitors represent the standard approach, with recent EBMT data showing no disadvantage from pre-transplant ruxolitinib exposure and better post-transplant outcomes in ruxolitinib-responsive patients than in those with no response or loss of response, underscoring the prognostic value of the depth of pre-transplant response [146]. Ruxolitinib should be tapered rather than abruptly discontinued around conditioning to avert the cytokine-release rebound and splenic re-enlargement associated with sudden withdrawal; the same molecule is a mainstay of treatment for both steroid-refractory acute and chronic GVHD after transplantation (REACH2 and REACH3) [147,148,149]. For patients with JAK-inhibitor-refractory splenomegaly, low-dose splenic irradiation is an emerging option. A recent EBMT retrospective analysis showed that pre-transplant splenic irradiation is feasible and effective in reducing spleen size, with acceptable toxicity and no negative impact on post-transplant outcomes [150].

The effect of the newer JAK inhibitors (fedratinib, momelotinib, pacritinib) on transplant outcomes remains undetermined.

### 5.2. Chronic Myeloid Leukemia

Tyrosine kinase inhibitors (TKIs) have collapsed allo-HSCT indications in chronic myeloid leukemia (CML) patients by more than 90% since 2000, and transplantation is not recommended in the frontline setting. The reference framework is the 2025 ELN recommendations [151], which incorporate the WHO 2022 reclassification of CML as a biphasic disease (chronic and blast phase ≥ 20% blasts, with abolition of accelerated phase) and replace the milestone terminology optimal/warning/failure with favorable/warning/unfavorable, reflecting evidence that ten-year survival with BCR::ABL1ᴵˢ in the range of 1–10% at 12 months remains around 80% and that reflexive switching is unwarranted in comorbid patients with modest residual disease. Six TKIs are now approved frontline, including the allosteric inhibitor asciminib, which gained first-line FDA approval in 2024 on the strength of ASC4FIRST (96-week major molecular response at 74.1% versus 52.0% for investigator-selected TKI, with lower-grade ≥3 toxicity) [152]. The T315I-directed inhibitor olverembatinib, approved in China, retains activity after both ponatinib and asciminib failure [153].

Allo-HSCT is indicated for chronic-phase CML that has failed at least three TKI generations, and for advanced-phase, blast-phase, or second-chronic-phase diseases. More granular ELN 2025 guidance recommends transplantation for chronic-phase patients on a third-generation or later TKI with BCR::ABL1 > 1% (or >0.1% with sustained molecular rise), for those intolerant to all available TKIs, and (with particular urgency) for T315I or compound mutations resistant to multiple TKIs [154]. Blast-phase patients should be treated with a TKI plus phenotype-adapted intensive chemotherapy (dasatinib- or ponatinib-based) and transplanted in a second chronic phase without needing to achieve a cytogenetic response e.g., MATCHPOINT) [155]. A practice-changing recommendation is to begin the donor search at failure of the first second-generation TKI, whether given first or second line, given the logistical delay in donor identification, and certainly at the emergence of high-risk additional chromosomal abnormalities or resistance mutations [156]. Contemporary outcomes are encouraging. An EBMT analysis of 904 chronic-phase patients found that OS and progression-free survival were determined by disease phase and Karnofsky score, and not by the number of prior TKI lines [157]. Myeloablative conditioning is preferred where feasible, and PTCy-based MMAD transplantation is a valid option when no matched donor is available. Treatment-free remission strategies (final EuroSKI analysis: 46% sustained a major molecular response at 12 months) continue to expand but do not affect transplant decisions [158].

Health–economic considerations, including the marked cost differential between generic imatinib and later-generation TKIs, further shape transplant decision-making in resource-constrained settings.

### 5.3. Polycythemia Vera and Essential Thrombocythaemia

Allo-HSCT is not indicated in polycythemia vera or essential thrombocythaemia unless the disease transforms to secondary myelofibrosis or to MDS/acute leukemia, in which case it follows the guidance for MF or blast-phase MPN, respectively, considering that the risk of mortality and morbidity in these conditions is exclusively related to thrombotic risk [15,118,159,160,161,162].

## 6. Chronic Lymphocytic Leukemia and Rare Mature Lymphoid Leukemias

The role of allo-HSCT in chronic lymphocytic leukemia (CLL) cases has been profoundly reshaped by covalent and non-covalent BTK inhibitors (ibrutinib, acalabrutinib, zanubrutinib, pirtobrutinib) and by venetoclax, used alone or in combination [163,164,165]. The ibrutinib–venetoclax combination improved progression-free and overall survival in the phase III FLAIR trial [166]. Consequently, allo-HSCT is now reserved (as a clinical option) for fit “double-refractory” patients who have exhausted both covalent/non-covalent BTK inhibitors and venetoclax-based regimens, particularly those with high-risk features (TP53 aberration, complex karyotype), whose survival without intervention is only in the range of 4–6 months [167,168].

Patients with clonally related Richter transformation should be considered for allo-HSCT regardless of CLL treatment stage, increasingly bridged with CD19 CAR-T, which is itself emerging as an option in double-refractory CLL [169,170,171]. Auto-HSCT is not recommended in CLL.

T-cell prolymphocytic leukemia retains a dismal prognosis (median survival of ~12 months with alemtuzumab) [172,173]. In fit patients responding to alemtuzumab, allo-HSCT from any donor is the only potentially curative intervention and should be pursued promptly [174].

## 7. Lymphomas

Since 2015, the EBMT registry has distinguished “true” CR1 (achieved directly by first-line therapy) from “first CR” attained after salvage of primary induction failure, sharpening prognostic segregation [175]. Across most lymphoma subtypes, retrospective analyses now show comparable outcomes for matched sibling, matched unrelated, and PTCy-based haploidentical allografts, allowing consistent recommendations [176].

### 7.1. Classical Hodgkin Lymphoma

Frontline ABVD, escalated BEACOPP, or the newer PET-adapted brentuximab vedotin–AVD and nivolumab–AVD schedules cure approximately 70–85% of patients [177,178]. For chemosensitive relapsed or primary refractory classical Hodgkin lymphoma patients, auto-HSCT after salvage remains the standard of care, grounded in the historical BNLI and HD-R1 randomized trials [179]. Modern salvage incorporating brentuximab vedotin with checkpoint inhibitors or chemotherapy raises pre-transplant complete metabolic response rates to a range of 70–90%, and brentuximab maintenance for 16 cycles after auto-HSCT improves progression-free survival in high-risk patients [180,181]. Allo-HSCT is reserved for relapse after a prior autograft, now typically third or fourth line after checkpoint-inhibitor exposure, with haploidentical PTCy transplantation having largely supplanted matched-alternative approaches [167,182]. CAR-T has not convincingly outperformed other modalities in this setting to date.

### 7.2. Large B-Cell Lymphoma

The management of large B-cell lymphoma (LBCL, encompassing diffuse large B-cell lymphoma, high-grade B-cell lymphoma, and primary mediastinal B-cell lymphoma) has been transformed by CD19 CAR-T and bispecific antibodies [183,184,185]. In primary refractory disease or relapse within 12 months of first-line therapy, the pivotal ZUMA-7 (axicabtagene ciloleucel) and TRANSFORM (lisocabtagene maraleucel) trials demonstrated the superiority of second-line CAR-T over salvage chemoimmunotherapy plus auto-HSCT, with a five-year OS benefit confirmed for axicabtagene ciloleucel [186,187,188]. By contrast, BELINDA with tisagenlecleucel was negative, probably reflecting a longer manufacturing interval [189]. Accordingly, CD19 CAR-T is the standard of care for primary refractory disease and early relapse with untested or chemoresistant disease, whereas auto-HSCT remains a valid option for early chemosensitive relapse and the standard for late (>12 months) chemosensitive relapse, a standard not yet challenged by a randomized trial [183,188]. In patients achieving CR after salvage before cell therapy, a retrospective analysis favored auto-HSCT over CAR-T consolidation, though the comparison was confounded by product and line-of-therapy differences [190]. After CAR-T failure, allo-HSCT remains a clinical option for eligible patients, and bispecific antibodies (epcoritamab, glofitamab) offer an increasingly used alternative [191,192,193]. For primary CNS lymphoma, auto-HSCT with thiotepa-based conditioning in first remission remains the standard for fit patients (including selected fit, older patients) and is preferred over whole-brain radiotherapy given the latter’s neurocognitive toxicity [194,195], with CAR-T emerging in the relapsed setting.

### 7.3. Follicular and Marginal-Zone Lymphomas

Most patients with advanced follicular lymphomas enjoy prolonged remissions after immunochemotherapy and maintenance, and should not receive cellular therapy in first remission. Auto-HSCT is a standard option for chemosensitive relapse, particularly with early progression within 24 months of first-line therapy (POD24), a validated adverse feature [196]. The treatment algorithm is shifting rapidly. Bispecific antibodies (mosunetuzumab, epcoritamab, odronextamab) are approved from third line, and two CD19 CAR-T products are approved from third/fourth lines [197]. Allo-HSCT, from a matched donor, is recommended for relapse after a prior autograft, especially early relapse, when CAR-T is unavailable [198]. Untransformed marginal-zone lymphoma is generally not a transplant indication [199]. Transformed follicular lymphoma is treated as large B-cell lymphoma.

### 7.4. Mantle Cell Lymphoma

The role of frontline auto-HSCT consolidation in mantle cell lymphoma has been challenged by the TRIANGLE trial, in which the addition of ibrutinib to induction with or without auto-HSCT yielded three-year failure-free survival rates of 88% (ibrutinib plus auto-HSCT) and 86% (ibrutinib without auto-HSCT) versus 72% for auto-HSCT alone, questioning the necessity of transplant in the BTK-inhibitor era [200]. In the salvage setting, auto-HSCT is an option for transplant-naïve patients who respond to chemoimmunotherapy, whereas brexucabtagene autoleucel is the standard of care for relapsed/refractory disease after BTK-inhibitor failure [201]. Allo-HSCT is confined to patients without access to, or failing, CAR-T [202].

### 7.5. Peripheral T-Cell Lymphoma

For the major nodal peripheral T-cell lymphoma (PTCL) entities, such as PTCL not otherwise specified, nodal T-follicular-helper lymphoma (formerly angioimmunoblastic) and ALK-negative anaplastic large-cell lymphoma, CHO(E)P-based induction followed by auto-HSCT consolidation in CR1 remains standard practice (NLG-T-01) [203], whereas frontline allo-HSCT is not indicated, the randomized AATT trial having shown no benefit over autograft [204]. ALK-positive anaplastic large-cell lymphoma does not require consolidation in low-risk patients [205]. For chemosensitive relapsed PTCL, allo-HSCT is the recommended consolidation whenever feasible, with brentuximab vedotin (for CD30-positive disease) or epigenetic combinations as bridging therapy [206]. Selected entities with dismal chemotherapy outcomes (hepatosplenic, monomorphic epitheliotropic intestinal, extranodal NK/T-cell, and adult T-cell leukemia/lymphoma) warrant allo-HSCT as first-line therapy [207]. Advanced-stage primary cutaneous T-cell lymphoma (mycosis fungoides/Sézary syndrome, EORTC/ISCL stages IIB–IV) patients benefit from the graft-versus-lymphoma effect of allo-HSCT, which improved progression-free survival over conventional therapy in a matched prospective study [208]. Auto-HSCT is not recommended because of high relapse rates.

## 8. Multiple Myeloma

### 8.1. Frontline Induction and the Role of Auto-HSCT

Frontline induction for transplant-eligible adults has shifted from triplet (bortezomib–lenalidomide–dexamethasone, VRd) to anti-CD38-containing quadruplet regimens. The PERSEUS trial (daratumumab–VRd versus VRd) demonstrated superior 48-month progression-free survival (84.3% versus 67.7%; HR 0.42) and far higher rates of MRD-negativity at 10^−5^, and IsKia established an analogous benefit for isatuximab–carfilzomib–lenalidomide–dexamethasone followed by auto-HSCT [209,210]. Despite these advances, single auto-HSCT consolidation in first-line therapy remains the standard of care for younger, fit patients, as confirmed by DETERMINATION (median progression-free survival at 67 versus 46 months for VRd plus auto-HSCT versus VRd alone, without an OS difference at current follow-up) and by the long-term IFM 2009 analysis [211,212,213]. Whether patients achieving MRD-negativity at 10^−6^ after quadruplet induction still require auto-HSCT is a major open question that the MIDAS and MASTER-2 trials are addressing; pending those results, auto-HSCT remains the standard. Total-body irradiation should not be used in conditioning, and the benefit of tandem auto-HSCT in the quadruplet era (historically reserved for high-risk cytogenetics) is no longer clear (EMN02/HO95) [214].

Beyond clinical efficacy, upfront auto-HSCT retains a favorable cost profile relative to indefinite exposure to anti-CD38-containing quadruplet regimens and subsequent BCMA-directed therapies, a consideration of particular weight in resource-constrained settings.

### 8.2. Salvage Transplantation, Allo-HSCT, and Cellular Therapies

Salvage auto-HSCT is an option for patients relapsing beyond 36 months after a first autograft with lenalidomide maintenance (or beyond ~18–24 months without maintenance), supported by randomized data showing a superior time to progression and a landmark OS signal in the ReLApsE trial [210,215]. Allo-HSCT in myeloma patients has curative potential but carries considerable non-relapse mortality and is now reserved (as a clinical option in experienced centers) for selected younger patients with high-risk disease, extramedullary relapse, or primary plasma cell leukemia, generally within trials [216,217]. PTCy has made allografting more feasible, though relapse remains the dominant challenge [218]. The decisive shift, however, has come from BCMA-directed cellular therapy. Ciltacabtagene autoleucel and idecabtagene vicleucel produced unprecedented responses in relapsed/refractory disease and, in CARTITUDE-4 and KarMMa-3, outperformed standard care in earlier lines [219,220], moving CAR-T into second/third lines. BCMA-, GPRC5D-, and FcRH5-directed bispecific antibodies (teclistamab, elranatamab, talquetamab, cevostamab) further displace allografting [221,222]. Consequently, allo-HSCT in myeloma cases is now a niche procedure for highly selected patients, with roughly 200 allografts reported to the EBMT in 2023.

### 8.3. AL Amyloidosis

Patients with systemic immunoglobulin light-chain (AL) amyloidosis without severe cardiac involvement may benefit from auto-HSCT, with careful risk assessment using cardiac-biomarker-based staging [223,224].

Early mortality has fallen substantially with appropriate patient selection, and the emerging efficacy of anti-plasma-cell antibodies and bispecifics is likely to further narrow the role of transplantation.

## 9. Bone Marrow Failure Syndromes and Non-Malignant Disorders

### 9.1. Acquired Severe Aplastic Anemia

HLA-identical sibling allo-HSCT is the standard front-line treatment for adults with severe aplastic anemia (SAA), with outcomes declining with age. Mortality becomes meaningful above 40 years and unacceptable above 50 years, so careful comorbidity assessment is essential in borderline age groups [225]. To limit chronic GVHD, all patients receive in vivo lymphodepletion with antithymocyte globulin (ATG) or alemtuzumab, and bone marrow is the preferred graft source; conditioning is age-adapted (high-dose cyclophosphamide in the young, fludarabine-based reduced-dose cyclophosphamide thereafter), without irradiation for sibling transplants [226,227]. First-line immunosuppressive therapy for patients lacking a sibling donor, or aged over 40 years, is now triple therapy with horse ATG, ciclosporin, and eltrombopag, the addition of eltrombopag having improved the complete response rate in the RACE trial [228,229]. Because triple therapy maximizes the response to immunosuppression, allo-HSCT from a matched unrelated donor or, increasingly, a mismatched alternative donor has become the preferred option for patients failing one course of immunosuppression, with recipient age, HLA disparity, and the interval from diagnosis to transplant (ideally within one year) governing the outcome [230]. Matched-unrelated-donor transplantation may even be a front-line option in young patients when a 10/10 donor is promptly available. For refractory/relapsed disease lacking a matched donor, haploidentical transplantation following the “DeZern” platform (fludarabine, cyclophosphamide, ATG and low-dose TBI with PTCy) yields excellent results and is a clinical option, while its use as initial therapy remains investigational [231]. A myeloid malignancy arising as clonal evolution (proven MDS or AML) mandates allo-HSCT, whereas the mere presence of somatic mutations does not.

### 9.2. Paroxysmal Nocturnal Hemoglobinuria

Complement inhibitors (the anti-C5 agents eculizumab and ravulizumab, the C3 inhibitor pegcetacoplan, and the oral factor B inhibitor iptacopan) have essentially eliminated allo-HSCT as front-line therapy in classical paroxysmal nocturnal hemoglobinuria cases [232]. Transplantation remains an option only for the aplasia/paroxysmal nocturnal hemoglobinuria syndrome with severe cytopenia (following SAA criteria), for evolution to a myeloid malignancy, or where complement inhibition is unavailable.

### 9.3. Constitutional Bone Marrow Failure, Pure Red Cell Aplasia, and Inherited Disorders in Adults

Constitutional bone marrow failure syndromes (Fanconi anemia, dyskeratosis congenita and other telomere disorders, Diamond–Blackfan anemia, Shwachman–Diamond syndrome) are increasingly present or require treatment in adulthood and mandate a dedicated diagnostic work-up, since the diagnosis dictates donor choice and conditioning [233]. Standard-dose chemotherapy and irradiation must be avoided in Fanconi anemia because of defective DNA repair [234]. Radiation-free fludarabine/low-dose cyclophosphamide regimens with a T-cell-depleted graft yield excellent results [235]. Telomere disorders require fludarabine-based reduced-intensity conditioning, and pre-transplant organ (lung, liver) damage predicts poorer outcome. Acquired pure red cell aplasia is managed medically (ciclosporin, rituximab, sirolimus), with transplantation as an exception [233].

For adults with hemoglobinopathies, allo-HSCT from a matched sibling donor is a curative option for selected patients with transfusion-dependent β-thalassaemia (in the absence of severe iron-related organ damage) and sickle cell disease (with severe clinical disease and absence of severe organ damage), and EBMT registry data now show that age is no longer an adverse determinant, with success rates exceeding 90% in adults [236]. Haploidentical transplantation with PTCy or T-cell-depleted platforms has extended eligibility. Since 2023–2024, gene therapy has provided a transformative alternative. CRISPR-Cas9-edited autologous CD34+ cells (exagamglogene autotemcel) are approved for both transfusion-dependent β-thalassemia and severe sickle cell disease, alongside lentiviral β-globin gene-addition products [237,238]. Adult primary immunodeficiencies (e.g., CTLA-4 haploinsufficiency, common variable immunodeficiency with enteropathy) are increasingly referred for allo-HSCT at specialist centers [233].

## 10. Transplant-Related Mortality, GVHD, GVL, and Future Directions

### 10.1. Non-Relapse Mortality

Over the past two decades, transplant-related mortality has fallen steadily, with a CIBMTR longitudinal analysis reporting a 52% reduction in 200-day non-relapse mortality relative to the 1990s [239,240,241]. Risk is stratified using the HCT-CI, the EBMT score, augmented comorbidity indices, and machine-learning models, which may further refine day-100 mortality prediction [44]. Infection prophylaxis has contributed materially: letermovir markedly reduces the incidence of clinically significant CMV infection, and mold-active azoles, together with preemptive monitoring for EBV and adenovirus, have reduced infectious mortality [242].

Advanced age no longer represents an absolute barrier to allo-HSCT. The BMT CTN 1102 prospective donor-versus-no-donor study extended the evidence base up to 75 years of age in higher-risk MDS, showing a survival benefit for RIC allo-HSCT over non-transplant strategies [101]. In septuagenarian patients, contemporary practice favors reduced-intensity or reduced-toxicity platforms (typically fludarabine–melphalan, treosulfan–fludarabine, or fludarabine–busulfan at reduced doses) coupled with PTCy-based GVHD prophylaxis, which further lowers early transplant-related mortality [101]. Careful geriatric assessment integrating the HCT-CI, functional status, and cognitive/nutritional reserve is now regarded as essential, particularly above the age of 70 years, and machine-learning models incorporating these variables may further refine individual risk prediction [44].

### 10.2. Acute and Chronic GVHD

Prophylactic strategies against graft-versus-host disease have shifted substantially, with post-transplant cyclophosphamide (PTCy) rapidly becoming the universal backbone. In the BMT CTN 1703 trial, PTCy/tacrolimus/mycophenolate proved superior to tacrolimus/methotrexate in reduced-intensity matched-donor transplantation [9]. Longer-term follow-up of BMT CTN 1703 has confirmed the durable benefit of PTCy-based prophylaxis, with sustained improvement in chronic GVHD-free, relapse-free survival [243]. The rationale for combining PTCy with a calcineurin inhibitor and mycophenolate mofetil rests on complementary mechanisms: PTCy selectively eliminates alloreactive donor T cells early after infusion, while calcineurin inhibition and mycophenolate provide sustained suppression during immune reconstitution [231,243]. Ongoing trials are testing whether the calcineurin inhibitor can be replaced by sirolimus, or whether the intensity of the PTCy/calcineurin/mycophenolate backbone can be de-escalated in lower-risk transplants, but none of these variants has yet supplanted the standard triple combination.

For 7/8 mismatched unrelated donors, abatacept reduces severe acute GVHD and is FDA-approved [244]. ATG remains the standard in European unrelated-donor transplants, with head-to-head data against PTCy still maturing. For steroid-refractory acute GVHD, ruxolitinib is standard of care, with a 28-day response of 62% versus 39% for best available therapy in REACH2 [149]. Moderate-to-severe chronic GVHD, which affects 30–50% of long-term survivors, now has three effective agents beyond corticosteroids: ruxolitinib in second-line (REACH3), and belumosudil (ROCKstar) and the anti-CSF1R antibody axatilimab in third-line therapies or beyond (AGAVE-201), the latter targeting the monocyte/macrophage compartment that drives sclerotic disease [147,245,246]. Updated efficacy and safety data from AGAVE-201 have confirmed durable responses across organ manifestations of chronic GVHD, supporting axatilimab as a new standard second-line option [246].

A further advance in GVHD prevention is the recent FDA approval of Orca-T, a precision-engineered allogeneic cell therapy product consisting of purified CD34^+^ hematopoietic stem cells combined with a defined dose of regulatory T cells and conventional T cells, administered after myeloablative conditioning. In the pivotal phase III Precision-T trial, Orca-T reduced acute and chronic GVHD and improved GVHD-free, relapse-free survival compared with conventional allo-HSCT with standard prophylaxis, without compromising engraftment or relapse control [247]. This product represents the first defined-composition graft to obtain regulatory approval in the transplant setting and illustrates the maturation of cellular engineering as a strategy to more cleanly separate GVL from GVHD.

A summary of strategies for GVHD management is presented in Table 2.

### 10.3. Preserving Graft Versus Leukemia

The central challenge is to suppress GVHD without abrogating GVL. Contemporary strategies include donor lymphocyte infusions for relapse or MRD re-emergence (with or without hypomethylating priming), pre-emptive hypomethylating agents guided by MRD (the RELAZA2 paradigm), targeted post-transplant maintenance (sorafenib and gilteritinib for FLT3-mutated AML; TKIs for Ph-positive ALL), and CAR-T as salvage for post-transplant relapse [56].

### 10.4. Health–Economic Considerations and HSCT in Low- and Middle-Income Countries

Cost is an increasingly relevant dimension of the transplant decision and one that varies dramatically across health systems. In high-income countries, the introduction of novel targeted therapies has substantially altered the cost balance between transplantation and non-transplant strategies. In multiple myeloma, upfront single auto-HSCT remains substantially less expensive than lifelong exposure to anti-CD38-containing quadruplet regimens and subsequent BCMA-directed cellular or bispecific therapies, an economic consideration that adds to its clinical rationale in fit patients, particularly in resource-constrained settings [248]. In CML patients, allo-HSCT was historically less costly than continued TKI therapy, a comparison substantially reshaped by the availability of generic imatinib (annual cost as low as a USD 200–500 range), which has made lifelong TKI economically feasible even in most LMIC settings, though second- and third-generation TKIs and asciminib remain financially prohibitive in many countries [151].

In the specific case of PTCy-based haploidentical transplantation, the standard 50 mg/kg/day dose on days +3 and +4 has been challenged by prospective and registry data suggesting that a reduced total dose (e.g., 25–40 mg/kg/day) may retain comparable GVHD-free, relapse-free survival with lower hematological and infectious toxicity, potentially reducing the length of hospitalization and downstream costs [249,250]. Confirmatory randomized data are awaited, but reduced-dose PTCy is of particular interest for LMIC settings where supportive-care resources are limited.

More broadly, HSCT activity is expanding rapidly in low- and middle-income countries, where haploidentical transplantation with PTCy has been transformative by removing the historical dependence on international unrelated-donor registries [251]. Reported outcomes from experienced LMIC centers now approach those of high-income centers, though at the cost of a higher burden of infectious complications, more limited access to novel GVHD-directed agents (ruxolitinib, belumosudil, axatilimab) and cellular therapies (CAR-T, bispecifics), and more restrictive donor-selection algorithms [252]. Reduced-intensity conditioning, use of bone marrow rather than peripheral blood in selected settings, extended CMV surveillance, and locally adapted supportive-care protocols are among the pragmatic adaptations that have made HSCT deliverable in these environments. In this context, the choice of transplant modality must integrate not only disease biology and patient fitness but also drug availability, cost, and local capacity for post-transplant surveillance and complications’ management.

### 10.5. Future Directions

Four trends define the future of this research. First, transplant selection is moving from static baseline risk to continuous, MRD-driven, response-adapted algorithms, with NPM1/FLT3 AML, Ph-positive ALL, MDS, and mantle cell lymphoma leading the way. Second, cellular therapies will continue to displace transplantation in aggressive B-cell lymphoma and myeloma cases while generating new indications, notably bridge-to-transplant and post-CAR-T consolidation in high-risk ALL patients. Third, graft engineering and tolerance induction (αβ-TCR/CD19-depleted grafts, selective regulatory T-cell strategies, and defined-composition products) aim to more cleanly separate GVHD from GVL. Fourth, gene therapy is becoming an established alternative to allo-HSCT for non-malignant disease, with CRISPR-edited and lentiviral autologous products now clinically available for sickle cell disease and β-thalassemia, and next-generation approaches advancing for congenital marrow failure syndromes and inborn errors of immunity.

## 11. Conclusions

HSCT remains a cornerstone of adult hematology, but its role is being redefined. Allo-HSCT remains central in high-risk acute leukemias, higher-risk MDS, and myelofibrosis, while auto-HSCT retains its role in relapsed Hodgkin lymphoma, PTCL, CNS lymphoma, and frontline myeloma. Elsewhere, CAR-T and bispecifics are progressively displacing transplantation. Looking ahead, several developments are likely to reshape practice further: CAR-T is increasingly deployed as a bridge to allo-HSCT in high-risk B-ALL and, conversely, as a potential replacement for transplantation in selected relapsed/refractory settings; gene-editing approaches (CRISPR-Cas9-based exagamglogene autotemcel) are already an established alternative to allo-HSCT for adult sickle cell disease and transfusion-dependent β-thalassemia, and next-generation gene therapies are advancing for congenital marrow failure syndromes and inborn errors of immunity. Driven by PTCy and MRD-guided decisions, the clinician’s question is no longer “transplant or not?”, but which donor, conditioning, bridge, and maintenance, tailored to disease biology, MRD trajectory, and patient fitness.

## Figures and Tables

**Table 1:** Synopsis of indications for hematopoietic stem-cell transplantation in adults, by disease and disease status. Grading follows the EBMT 2025 four-tier system (adapted from Greco R et al., Bone Marrow Transplant 2025 [15]). S: standard of care; CO: clinical option; D: developmental; GNR: generally not recommended; (–): not applicable. MSD/MUD: matched sibling or well-matched (10/10) unrelated donor; MMAD: mismatched alternative donor (9/10; mismatched unrelated, haploidentical, or cord blood); Auto: autologous HSCT; MRD: measurable residual disease.

Disease	Disease Status	MSD/MUD Allo	MMAD Allo	Auto
AML	CR1, favorable risk, MRD–	GNR	GNR	GNR
AML	CR1, favorable risk, MRD+	S	CO	GNR
AML	CR1, intermediate risk	S	CO	CO
AML	CR1, adverse risk	S	S	GNR
AML	CR2	S	S	CO
APL	Molecular CR2	S	CO/GNR	S
ALL	Ph–, CR1, standard risk, MRD–	GNR	GNR	GNR
ALL	Ph–, CR1, standard risk, MRD+	S	CO	GNR
ALL	Ph–, CR1, high risk	S	CO	GNR
ALL	Ph+, CR1, MRD–	S	CO	CO
ALL	Ph+, CR1, MRD+	S	S	GNR
ALL	CR2/relapsed–refractory (bridged)	S	S/CO	GNR
CML	Chronic phase, failing ≥3 TKI	S	CO	GNR
CML	Advanced/blast phase/>CP1	S	S	GNR
Myelofibrosis	Primary Int-2/high (DIPSS) or high MIPSS70; secondary high/Int-2 MYSEC-PM	S	S	GNR
MDS	Very low/low (IPSS-R/IPSS-M)	GNR	GNR	GNR
MDS	Higher risk (IPSS-R Int-2/high; IPSS-M mod–high/high/very high)	S	S	GNR
CMML	CPSS-Mol high (or Int-2 + risk factor)	S	S	GNR
CLL	Double refractory to BTKi + BCL2i, high risk	CO	CO	GNR
CLL	Richter transformation (clonally related)	S	S	GNR
LBCL	Primary refractory/early relapse (≤12 mo)	GNR	GNR	GNR
LBCL	Late chemosensitive relapse ≥ CR2	GNR	GNR	S
LBCL	Relapse after auto-HSCT/after CAR-T	CO	CO	–
Follicular	Chemosensitive relapse ≥ CR2 after auto-HSCT	S	CO	GNR
Mantle cell	CR1	GNR	GNR	S/CO
Mantle cell	Relapsed/refractory after BTKi	CO	CO	GNR
PTCL	CR1	–	–	S/CO
PTCL	Chemosensitive relapse ≥ CR2/PR2	S	CO	CO
Hodgkin	Chemosensitive relapse, no prior auto-HSCT	GNR	GNR	S
Hodgkin	Chemosensitive relapse after auto-HSCT	S	S	CO
Multiple myeloma	Upfront (standard/high risk)	CO	GNR	S
Multiple myeloma	R/R after ≥3 lines (IMiD + PI + anti-CD38)	–	–	–
AL amyloidosis	Without severe cardiac involvement	–	–	CO
Severe aplastic anemia	Newly diagnosed (age ≤ 40 years)	S	CO	–
Severe aplastic anemia	Relapsed/refractory	S	S/CO	–
PNH	AA/PNH syndrome (severe cytopenia)	CO	CO	–
β-thalassemia (TDT)	No severe organ damage	CO	CO	–
Sickle cell disease	Severe, no severe organ damage	S	CO	–

**Table 2:** Contemporary prophylaxis and treatment of graft-versus-host disease (GVHD).

Setting	Agent/Regimen	Outcome and Notes
Prophylaxis		
Matched donor (RIC/MAC)	Post-transplant cyclophosphamide (PTCy) + tacrolimus + mycophenolate mofetil	Superior 1-year GVHD-free, relapse-free survival (52.7% vs. 34.9%; HR 0.64) vs. tacrolimus/methotrexate; now a standard backbone across donor types
Haploidentical donor	PTCy-based	Outcomes comparable to 10/10 MUD; has widened the donor pool
Mismatched unrelated donor (MMUD)	PTCy-based	Feasible and effective; extends access for patients lacking a matched donor
7/8 mismatched unrelated donor	Abatacept added to calcineurin inhibitor + methotrexate	Reduces grade III–IV acute GVHD; FDA-approved for this setting
Unrelated donor (European practice)	Antithymocyte globulin (ATG)	Reduces chronic GVHD; head-to-head data against PTCy still maturing
Acute GVHD		
First line	Corticosteroids (methylprednisolone/prednisolone 1–2 mg/kg/day)	~50% respond; steroid-refractory disease carries a poor prognosis
Steroid refractory	Ruxolitinib (JAK1/2 inhibitor)	28-day overall response 62% vs. 39% for best available therapy; standard of care
Chronic GVHD		
First line	Corticosteroids ± calcineurin inhibitor	Affects 30–50% of long-term survivors; leading cause of late morbidity
Second line	Ruxolitinib (JAK1/2 inhibitor)	Superior overall response and failure-free survival vs. best available therapy
Third line or beyond	Belumosudil (ROCK2 inhibitor)	Overall response ~74%; effective in heavily pre-treated disease
Third line or beyond	Axatilimab (anti-CSF1R antibody)	Targets the monocyte/macrophage compartment driving sclerotic disease; FDA-approved (2024)

Abbreviations: CSF1R, colony-stimulating-factor-1 receptor; GVHD, graft-versus-host disease; HR, hazard ratio; JAK, Janus kinase; MAC, myeloablative conditioning; MUD, matched unrelated donor; PTCy, post-transplant cyclophosphamide; RIC, reduced-intensity conditioning; ROCK2, Rho-associated coiled-coil-containing protein kinase 2.

## Data Availability

No new data were created or analyzed in this study. Data sharing is not applicable to this article.
